# Investigating Differential Dynamics of the MAPK Signaling Cascade Using a Multi-Parametric Global Sensitivity Analysis

**DOI:** 10.1371/journal.pone.0004560

**Published:** 2009-02-23

**Authors:** Jeongah Yoon, Thomas S. Deisboeck

**Affiliations:** Complex Biosystems Modeling Laboratory, Harvard-MIT (HST) Athinoula A. Martinos Center for Biomedical Imaging, Massachusetts General Hospital, Charlestown, Massachusetts, United States of America; Center for Genomic Regulation, Spain

## Abstract

Cell growth critically depends on signalling pathways whose regulation is the focus of intense research. Without utilizing *a priori* knowledge of the relative importance of pathway components, we have applied *in silico* computational methods to the EGF-induced MAPK cascade. Specifically, we systematically perturbed the entire parameter space, including initial conditions, using a Monte Carlo approach, and investigate which protein components or kinetic reaction steps contribute to the differentiation of ERK responses. The model, based on previous work by Brightman and Fell (2000), is composed of 28 reactions, 27 protein molecules, and 48 parameters from both mass action and Michaelis-Menten kinetics. Our multi-parametric systems analysis confirms that Raf inactivation is one of the key steps regulating ERK responses to be either transient or sustained. Furthermore, the results of amplitude-differential ERK phosphorylations within the transient case are mainly attributed to the balance between activation and inactivation of Ras while duration-differential ERK responses for the sustained case are, in addition to Ras, markedly affected by dephospho-/phosphorylation of both MEK and ERK. Our sub-module perturbations showed that MEK and ERK's contribution to this differential ERK activation originates from fluctuations in intermediate pathway module components such as Ras and Raf, implicating a cooperative regulatory mode among the key components. The initial protein concentrations of corresponding reactions such as Ras, GAP, and Raf also influence the distinct signalling outputs of ERK activation. We then compare these results with those obtained from a single-parametric perturbation approach using an overall state sensitivity (OSS) analysis. The OSS findings indicate a more pronounced role of ERK's inhibitory feedback effect on catalysing the dissociation of the SOS complex. Both approaches reveal the presence of multiple specific reactions involved in the distinct dynamics of ERK responses and the cell fate decisions they trigger. This work adds a mechanistic insight of the contribution of key pathway components, thus may support the identification of biomarkers for pharmaceutical drug discovery processes.

## Introduction

Deregulated cell growth, a hallmark of cancer, is associated with perturbed signal transduction [1]. In response to external stimuli by specific ligands, receptor tyrosine kinases (RTKs) can alter cellular phenotypes such as cell survival, proliferation and migration [2]. There have been a number of different intracellular signaling pathways activated by RTKs, including epidermal growth factor (EGF) signaling [3]. The output of the signal transduction frequently targets the activation of the extracellular signal-regulated kinase (ERK), which is known to be involved in solid tumor formation by regulating cell cycle progression [4], [5]. Previous experimental studies support differential duration of ERK activity as being critical for cell signaling decisions [6]–[11]. However, it still remains uncertain which mechanisms control those phenotypic decisions. Thus, further characterizing the sub-cellular reaction steps or protein molecules that are critical for differential control of cellular activities is important not only to better understand the dynamics of complex signaling system for predictive purposes, but also to identify potential therapeutic targets for drug development [12].

The continuous advancement of high-throughout technologies in the post genomic era presents the challenge of how to interpret an ever growing amount of molecular data. Numerous experimental works have attempted to identify particular signaling molecules and their mechanisms, for example, by constructing mutants, overexpression, or reconstitutions of arrangement of genes or proteins [6]. Using experimental techniques alone, however, will make it difficult to identify decisive reaction steps or molecules that control for instance differential ERK responses. This is not only due to the inherently complex structure and function of various signal transduction pathways, but also because of marked nonlinearity within the system. Thus, it is useful to introduce computational, data- or hypothesis-driven approaches in an effort to facilitate the discovery of cell signaling decision factors [13]. Also, complex signaling pathways can be treated as a tightly connected network that orchestrates regulation of a specific functional behavior, rather than as individual, separate mechanisms by focusing on a specific oncogenic molecule or its activating event alone [14].

In this paper, we therefore apply a systems-level, multi-parametric perturbation strategy using a Monte Carlo (MC) simulation to discover molecules or reaction steps that orchestrate differential mitogen-activated protein kinase (MAPK) signaling responses. The model system is an EGF-induced signaling pathway, originally compiled by Brightman and Fell [15]. We have disturbed every single parameter without *a priori* knowledge on the relative importance of certain parameters and have generated massive samples of multiple perturbations for all parameters using our MC simulation; the peak amplitude (height) and duration (width) of ERK profiles (e.g., *transient vs. sustained*) are then used as differentiation measures [16], [17]. Our analysis reveals the dominant role of intermediate module proteins such as Ras and Raf as key controlling factors for the distinct dynamics of ERK activation. Although MEK and ERK in the MAPK module also showed sensitivities, they *alone* did not affect differential ERK dynamics without the co-perturbation of intermediate module proteins. This may implicate a *cooperative* regulation mode of key components in cellular responses. In addition, initial concentrations of the key proteins in corresponding reactions are also actively involved in determining the cellular responses. Lastly, we note that here identified critical molecules have already been experimentally validated as biomarkers.

## Methods

### EGFR Cell Signaling Model

**Figure 1:**
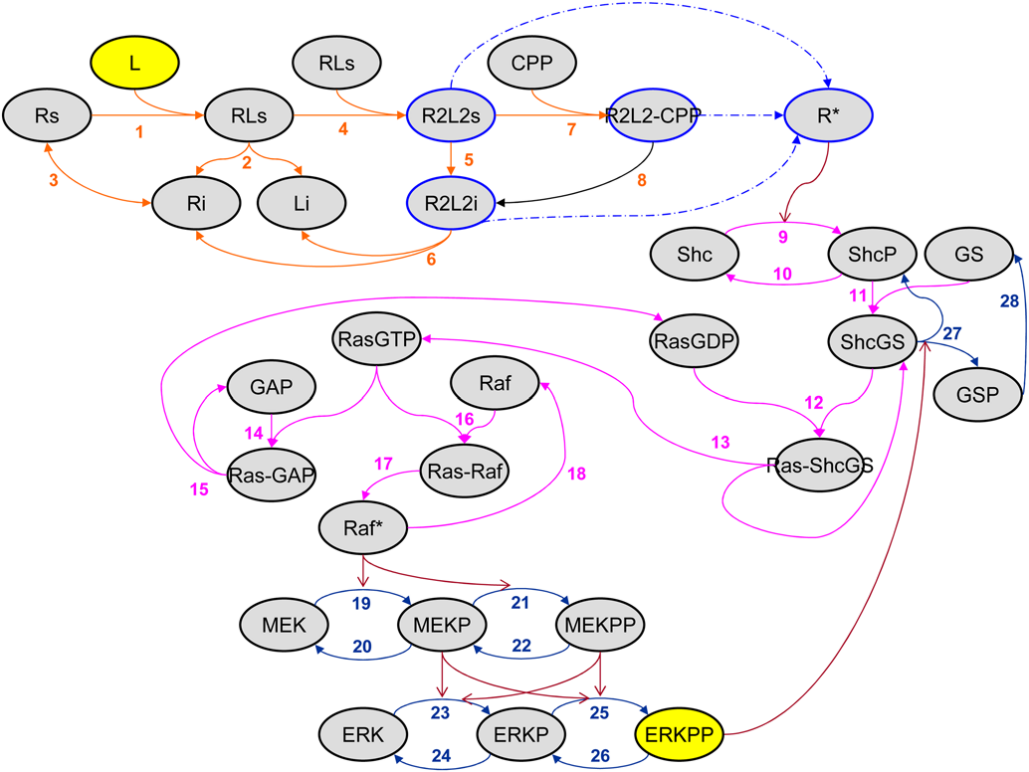
Schematic view of the EGF-R signal transduction pathway [15]. Top-level, intermediate, and MAP kinase modules are colored in *orange*, *magenta*, and *navy*, respectively. Filled arrowheads represent protein-protein binding reactions whereas open arrowheads denote catalytic interactions. Rs, free cell surface receptor; L, ligand; Ri, internal receptor; Li, internal ligand; RL, cell surface R-L complex; R2L2s, cell surface dimmer complex of RL; R2L2i, internalized dimmer complex of RL; CPP, coated pit protein; R2L2-CPP, receptor-ligand-coated pit protein complex; R*, all active species of R, i.e., R2L2s, R2L2i, and R2L2-CPP; Shc, Src homology and collagen domain protein; ShcP, phosphorylated Shc; GS, complex of Grb2, growth factor receptor binding protein 2, and SOS, Son of sevenless homologue protein; GSP, phosphorylated GS; ShcGS, complex form of Shc and GS; Ras-GDP, inactive, GDP-bound Ras; Ras-ShcGS, complex form of Ras-GDP and ShcGS; Ras-GTP, active, GTP-bound Ras; GAP, Ras GTPase activating protein; MEKP and MEKPP, phosphorylated forms of MEK; ERKP and ERKPP, phosphorylated forms of ERK.

The EGF receptor (EGFR) system implemented is based on the Brightman and Fell model (**Figure 1**) [15]. Although this pathway representation is moderately-sized, it covers the major cascade of an EGF-induced Ras-dependent MAP kinase signaling pathway with one feedback loop. Here, we provide a brief biological description of the signaling cascade. This network is divided into *three subsystems* according to somewhat separable roles and topographic locations.

The mechanisms in the *first* or top module occur close to the plasma membrane, and are related to the activation of the EGF receptor. First, the corresponding ligand EGF (L) as the only external stimulus binds to a monomeric receptor (R), forming an RL complex prior to being dimerized; at this point, intrinsic protein tyrosine kinases are activated. Only the activated dimer complex species are internalized through binding to cell-surface coated pit adaptor proteins (CPP). These complexes are then dissociated and degraded, and the monomeric species are recycled to the plasma membrane.

In the *second* or intermediate module, the activated receptor (R*) catalyzes the adaptor protein Src homology and collagen domain protein (Shc). Phosphorylated Shc (ShcP) then forms a ternary complex, ShcGS, with a constitutive complex of growth factor receptor binding protein 2 (Grb2) and Son of sevenless homologue protein (SOS), the guanylnucleotide exchange factor. Subsequently, the ShcGS complex recruits cytoplasmic SOS to the membrane-bound Ras protein, where the inactive Ras-GDP is activated to Ras-GTP through interaction with ShcGS. There are two downstream options with regards to active Ras-GTP: it either binds to GAP (GTPase-activating protein) to stimulate the GTPase activity of Ras so that RasGTP is converted to inactive RasGDP; or, Ras-GTP binds to Raf, forming the Ras-Raf complex such that Raf is recruited to the plasma membrane before the complex facilitates the Raf kinase activation (Raf*). The activated Raf in the latter option phosphorylates the mitogen-activated protein kinase (MAPK) cascade, which constitutes the third module.

In this *third* or MAPK module, both MEKP and MEKPP activate ERK by phosphorylating a tyrosine and a threonine residue, but only ERKPP is known to be active. ERKPP leads to the dissociation of the ShcGS complex through feedback regulation [18]. Raf*, MEKP, MEKPP, and ERKPP are dephosphorylated by the same phosphatase, protein phosphatase 2A (PP2A). (We note that there have been several studies that focused on understanding the dynamics of phospho-/dephosphorylation of this MAPK cascade module by combining *in silico* modeling with experimental data [19]–[23]).

### Multi-Parametric Global Sensitivity Analysis

#### Parameter Sensitivity Analysis based on a Monte Carlo Simulation

The EGFR signaling cascade system implemented here consists of 28 kinetic reactions involving 27 different protein molecules and 48 parameters. In general, these reactions follow mass action kinetics except for those catalyzed by enzymes, which follow Michaelis-Menten (MM) kinetics. The main goal of procedures detailed in this section is to calibrate key pathway elements (e.g., parameters, molecules, or reaction structures) that are chiefly responsible for processing cellular phenotypic decisions within a tolerable range. To this end, any approaches may face the following two challenges: First, even for a moderately sized cell signaling network, it is non-trivial to track the molecules' temporal behavior as kinetic parameters are often still unknown and difficult to measure experimentally. Second, even for known parameters, detailed quantitative measurements of protein activities may have been conducted under experimental condition that are far from the realities of an *in vivo* environment and that vary between experiments [24]. Thus, one may have to rely on potentially inaccurate measurements of input parameter sets.

We have therefore applied an approach that can avoid heavy dependence on a given set of initial parameter values. At the core is a Monte Carlo (MC) simulation that explores certain ranges of the parametric space around a given initial parameter value and generates samples of numerous parameter vectors. These vectors contain a set of multiply perturbed individual elements and are randomly generated using uniform distribution functions within known ranges of parameter uncertainty. This MC simulation is used for the entire parametric uncertainty analysis (see **Figure S1**). The detailed procedure for our MC simulation-based global uncertainty analysis is as follows [25], [26]:

#### Step 1

Define a range for *k* parameters (or every initial condition) involved in the signaling cascade, reflecting the uncertainties of signaling responses. The lower and upper bound of ranges has been suggested by [27], which reflects a variation of approximately (+/−) 2 orders of magnitude for the initial value of each parameter.

#### Step 2

Generate a series of independent random numbers using a uniform distribution for each parameter within defined ranges of uncertainties at Step 1. The total number of generated samples (N = 10,000) is assumed to be independent of each other and also sufficiently large in number.

#### Step 3

Run the ordinary differential equation (ODE) model for each set of *k* parameters and calculate an objective function value for the ERK profile. The objective function is defined as the sum of the squared errors of the active ERK level between the unperturbed (*unper*) and the perturbed (*per*) system as follows:
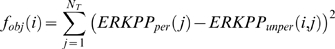
(1)where *j* is the number of time points and *i* is the total number of samples generated by the MC simulation (*i* = 1, …, N, N = 10,000).

#### Step 4

Compare the objective function value to a threshold value. In this study, the threshold is defined as the sum of the squared errors between the active ERK profile from the unperturbed system and the average active ERK profile from all samples. Based on the threshold, each parameter set is classified into either a tolerable sample group (*m* samples) when the error sum of the ERK signal from a certain parameter set is below the threshold, or an intolerable sample group (*n* samples) when it is above the threshold. Using the threshold, 99% of the generated samples have been classified into the tolerable group. We only retain the tolerable group samples and discard the others. Note that the sum of *m* and *n* is equal to N, the total number of samples generated by the MC method.

#### Step 5

**Figure 2:**
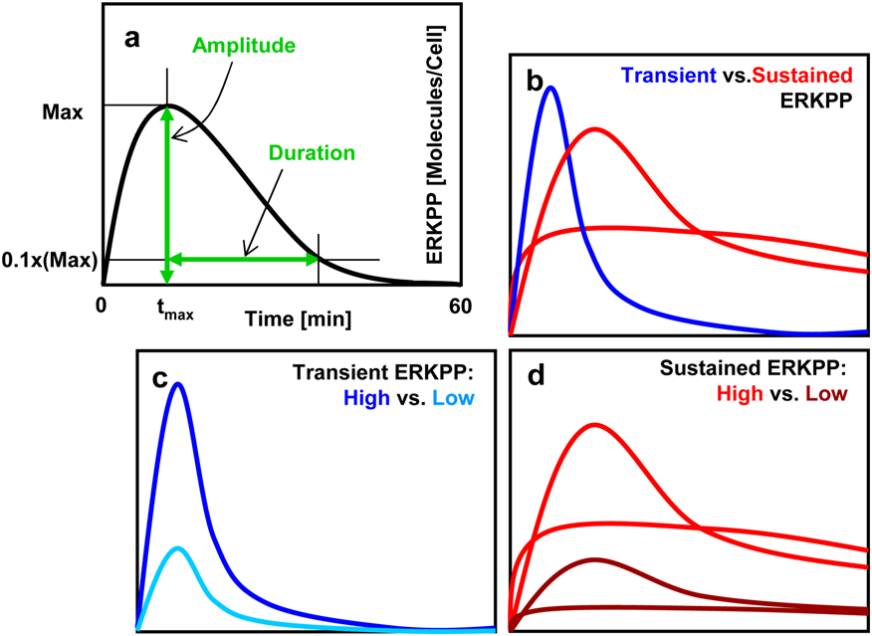
Transient and sustained time profiles of ERK activation. a) definitions of amplitude and duration (the ‘amplitude’ is defined as the maximum level of ERK over a time period of 60 min, and the ‘duration’ as the time period from the point of the maximum ERK level to the point of reaching 10% of the maximum within 60 min), b) transient ERK level (T) vs. sustained level (S), c) lowly transient ERK level (L-T) vs. highly transient level (H-T), and d) lowly sustained ERK level (L-S) vs. highly sustained level (H-S).

Distinguish differential profiles of ERK responses using tolerable group samples only. In this study, we consider three cases of two possible differential ERK responses: **i)** transient ERK level (T) vs. sustained level (S), **ii)** lowly transient ERK level (L-T) vs. highly transient level (H-T), and **iii)** lowly sustained ERK level (L-S) vs. highly sustained level (H-S). In order to classify samples of the tolerable group into the two types for each case, we introduce two characteristic measures, i.e., *amplitude* and *duration* of the ERK profile. In this study, we define the ‘amplitude’ as the maximum level of ERK over a time period of 60 min and the ‘duration’ as the time period from the point of the maximum ERK level to the point of reaching 10% of the maximum, within 60 min (**Figure 2**).

In order to efficiently classify and collect samples from the tolerable sample group for each case, we first sorted the samples with the maximum amplitude of ERK in ascending order. Then, for case 1 (T vs. S), transient samples are collected as those satisfying the criterion that the ERK level at the last time-point observation (i.e., at 60 min) is less than 10% of the maximum amplitude; sustained samples are collected according to the maximum duration, in addition to considering the maximum amplitude. For case 2 (L-T vs. H-T), L-T group samples are those below the median profile of ERK in case 1; H-T samples are those above the median. For case 3 (L-S vs. H-S), we further extracted samples with the duration of more than 30 min from the sorted samples with the maximum amplitude level in case 1. Because the maximum amplitude of ERK often occurs within the first 10 to 20 min (within the 60 min period), we assumed sustained samples would have the duration of more than 30 min; accordingly, samples of the duration of less than 30 min have been discarded. From the extracted sample list (ordered from the sample with the longest duration to that with the shortest) we have collected L-S samples from the bottom (shortest duration sample) of the list, while H-S samples have been taken from the top (longest duration sample) of the sample list. Selected were 367 samples for T and 500 samples for S in case 1, 365 samples for L-T and 367 samples for H-T in case 2, and 100 samples for both the L-S and H-S in case 3. Note that the number of samples for each group is arbitrarily chosen. During the process, our goal was that collected samples for each case have distinctively separable characteristics, so that results from the multi-parametric global sensitivity analysis can provide recognizable features for each comparison.

#### Step 6

Evaluate parametric sensitivities by comparing the parameter distributions between two sample sets of differential ERK responses for all three cases (i.e., T vs. S, L-T vs. H-T, and L-S vs. H-S). Here, we have simply calculated cumulative frequency (CF) distributions to identify informative parameters and reactions that contribute to the difference between two differential responses. For instance, if the CF distributions between the two groups for a certain parameter are distinctively different, i.e., yielding low correlation coefficients between the two CF distributions, the parameter is classified as a sensitive, fragile, or informative factor because it contributes to the control of a particular type of differential ERK responses; otherwise, it is classified as an insensitive, robust, or uninformative factor.

In the following section, we briefly introduce overall state sensitivities (OSSs) [28]. OSS is obtained by perturbing one *single* parameter at a time while keeping all other parameters fixed. This is in contrast to our MC-based approach that changes *multiple* parameters simultaneously. We will then analyze how the single parametric global sensitivity analysis can strengthen or weaken the results from the multi-parametric approach.

### Overall State Sensitivity Analysis

The OSS index is often used to capture global robustness of state variables upon parameter perturbations [29]. For example, in a simple enzyme mediated reaction, let the Michaelis constants *Km* be a parameter *p*, and the concentration of the substrate or activation level of the protein be a variable *X*. Then the parameter sensitivity of *X* regarding *p* is defined by
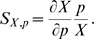
(2)


With this general definition in mind, to calculate the overall state sensitivity for the individual element *j* of a perturbed parameter set, the OSSs are integrated over discrete time 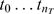 as described previously [28]:
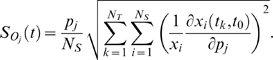
(3)


Here, N_T_ represents the number of time points while N_S_ denotes the number of protein molecules or protein complexes in the system. OSS describes how robust a system is to a single parameter change while the other parameters are fixed. We perturbed at most (+/−) 50% of the original parameter value. Note that all parameter sensitivities are only valid in a local space, i.e., within the proximal space of the unperturbed parametric space.

All numerical simulations of biochemical reaction-ODEs and MC-based simulations were implemented in MATLAB (version 7.5.0, MathWorks, Natick, MA). We used *ode15s* function for solving the nonlinear ordinary differential equations with 300 time points for a 60 min simulation. Using a workstation with a 2.00 GHz CPU and 4.00 GB of RAM it took approximately 3.5 hours of CPU time to simulate 10,000 samples for the entire pathway run.

## Results

The nominal parameter set, initial conditions, and their perturbed ranges are depicted in **Table 1** and **Table 2**. Note that the nominal parameter set of the EGFR model gives a transient pattern of ERK activity. In this section, we show results of comparing the aforementioned three cases: **i)** transient vs. sustained ERK profiles, **ii)** lowly transient vs. highly transient ERK profiles, and **iii)** lowly sustained vs. highly sustained ERK profiles. For each case, we examine which pathway components influence the differential responses most.

**Table 1:** Ranges for kinetic parameters used for the MC simulation [27].

Parameter [unit]	Parameter Value	Test Range	*Actual Test Range
k1 [M^−1^ min^−1^]	3.8e8	7.3e7–7.3e9	7.3518e7–7.2998e9
k2,	0.7,	0.1–1	0.1001–0.9997,
k5,	0.35,		0.1002–0.9999,
k6,	0.35,		0.1000–0.9999,
k8 [min^−1^]	0.35		0.1001–0.9999
k3 [min^−1^]	4.84e-2		
k4 [molecule^−1^min^−1^]	1.383e-3	1.8e-4–1.8e-2	1.8066e-4–1.8e-2
k7 [min^−1^]	1	0.1–10	0.1004–9.9989
k9 [min^−1^]	12	6.0–60	6.0003–59.9861
k11 [molecule^−1^min^−1^]	2.0e-3	2.0e-3–2.0e-1	2.0e-3–2.0e-1
k12 [molecule^−1^min^−1^]	1.63e-2	2.5e-5–6.0e-2	3.1199e-5–6.0e-2
k13 [min^−1^]	15	1.2–2.4e2	1.2045–2.3995e2
k14 [molecule^−1^min^−1^]	5.0e-3	5.0e-4–5.0e-2	5.0141e-4–5.0e-2
k15 [min^−1^]	7.2e2	3.0e2–1.2e3	3.0015e2–1.2e3
k16 [molecule^−1^min^−1^]	1.2e-3	1.2e-4–1.0e-2	1.2066e-4–1.0e-2
k17 [min^−1^]	27	0.15–2.4e2	0.1694–2.3994e2
k19,	50,	1.5–2.4e2	1.5210–2.3997e2,
k21 [min^−1^]	50		1.5313–2.3999e2
k23,	8.3,	1.45–2.4e2	1.4668–2.3998e2,
k25 [min^−1^]	8.3		1.4829–2.3999e2
k27 [min^−1^]	1.6	1.4–1.2e2	1.4088–1.1999e2
k_1 [min^−1^]	0.73	1.0e-10–1.0e-8	1.0052e-10–9.9991e-9
k_3 [min^−1^]	0.7	0.1–1	0.1001–0.9997
k_7 [min^−1^]	3.47e-4	3.47e-5–3.47e-3	3.5466e-5–3.5e-3
k_11 [min^−1^]	3.8		
k_12 [min^−1^]	10		
k_14 [min^−1^]	60		
k_16 [min^−1^]	3		
V10,	3.0e5,	0.6–3.0e6	854.6165–2.9988e6,
V18,	9.7e4,		66.7873–2.9992e6,
V28 [molecules cell^−1^ min^−1^]	75		276.2419–2.9999e6
V20,	9.2e5,	3.6e2–1.8e9	1.8663e5–1.7998e9,
V22,	9.2e5,		8.9270e4–1.7995e9,
V24,	2.0e5,		4.8764e5–1.7999e9,
V26 [molecules cell^−1^ min^−1^]	4.0e5		2.2472e5–1.7999e9
Km9,	6.0e3,	6.0e3–9.0e6	1.0976e4–8.9994e6,
Km10,	6.0e3,		6.0848e3–8.9980e6,
Km18 [molecules cell^−1^]	6.0e3		8.0619e3–8.9990e6
Km19,	9.0e3	6.0e3–9.0e6	6.0990e3–8.9999e6,
Km21,			9.1468e3–8.9997e6,
Km23,			6.4348e3–8.9996e6,
Km25 [molecules cell^−1^]			7.3475e3–8.9959e6
Km20,	6.0e5	6.0e3–9.0e6	6.2941e3–8.9983e6,
Km22,			6.7470e3–9.0e6,
Km24,			7.1524e3–8.9997e6,
Km26,			6.0566e3–8.9995e6,
Km27 [molecules cell^−1^]			9.8755e3–8.9999e6
Km28 [molecules cell^−1^]	2.0e4	6.0e3–9.0e6	6.4206e3–8.9995e6

* The column of actual test range represents parameter ranges that are contained in the generated samples.

**Table 2:** Ranges for initial conditions used for the MC simulation [27].

Initial Condition [unit]	Initial Value	Test Range	*Actual Test Range
x_0_1 [L_0_, M]	1.0e-7	2.0e-13–1.0e-7	2.3588e-11–9.9988e-8
x_0_2 [R_s0_, molecules cell^−1^]	1.11e4	1.0e4–1.0e6	1.0191e4–9.9996e5
x_0_4 [R_i0_, molecules cell^−1^]	3.9e3	1.0e3–1.0e5	1.0001e3–9.9992e4
x_0_9 [Shc_0_, molecules cell^−1^]	3.0e4	1.0e4–2.0e5	1.0009e4–1.9999e5
x_0_11 [GS_0_, molecules cell^−1^]	2.0e4	1.0e4–2.0e5	1.0002e4–1.9999e5
x_0_14 [RasGDP_0_, molecules cell^−1^]	1.98e4	1.0e4–1.0e6	1.0119e4–9.9998e5
x_0_16 [RasGTP_0_, molecules cell^−1^]	2.0e2	1.0e2–1.0e4	1.0049e2–9.9996e3
x_0_17 [GAP_0_, molecules cell^−1^]	1.5e4	1.0e4–2.0e5	1.0021e4–1.9995e5
x_0_18 [Raf_0_, molecules cell^−1^]	1.0e4	1.0e4–1.0e6	1.0201e4–9.9988e5
x_0_22 [MEK_0_, molecules cell^−1^]	3.6e5	1.0e4–1.0e6	1.0247e4–9.9994e5
x_0_25 [ERK_0_, molecules cell^−1^]	7.5e5	1.0e4–1.0e6	1.0024e4–9.9991e5

* The column of actual test range represents ranges of initial values that are contained in the generated samples.

### What are the most informative reactions that control transient vs. sustained ERK responses (i.e., T vs. S)?

**Figure 3:**
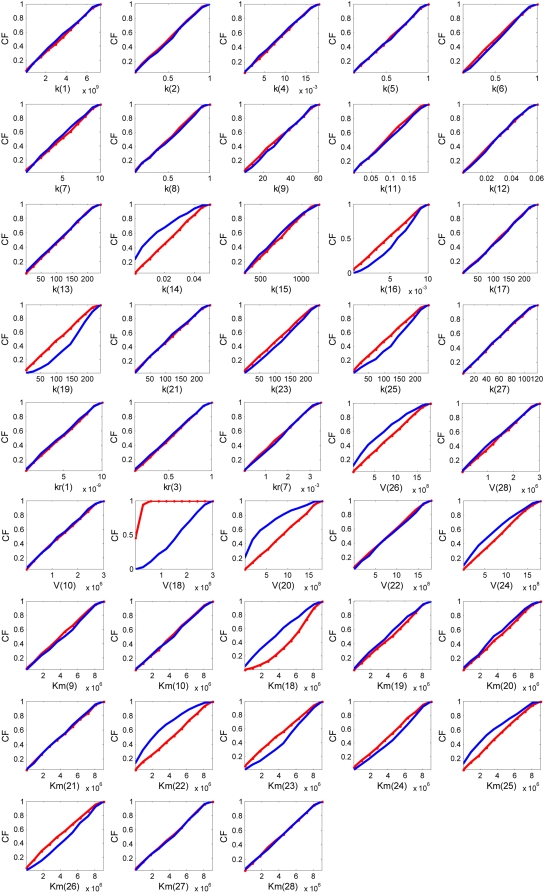
Results of cumulative frequency distributions between the sustained (S) and the transient (T) case of the multi-parametric sensitivity analysis for the whole EGFR network. Solid lines in *red* and in *blue* represent the S and T case, respectively.

First, we investigated the differentiating pathway parameters between transient and sustained activation of ERK profiles. The cumulative frequency (CF) distributions of transient and sustained cases for each parameter are shown in **Figure 3**; correlation coefficients between the two CF distributions are shown in **Table S1**, where larger correlation coefficients represent more robust parameters.

The multi-parametric analysis based on the MC method identifies k14, k16, k19, and k25 as the most sensitive forward mass-action kinetic parameters while no significant effects result from reverse kinetic parameters. Furthermore, the reaction rates of V18, V20, and mildly V24 and V26 appear to be closely involved in controlling the differential ERK responses. Finally, with regards to the Michaelis constants, Km18 and Km22–Km26 are the dominating parameters. Together, this indicates that the reaction steps R14, R16, R18, R19–R20, R22, R24, and R26 are those primarily controlling differential ERK responses (i.e., a transient vs. a sustained activation profile). The corresponding protein molecules involved in those reactions include active Ras and Raf (RasGTP and Raf*), MEK, and ERK.

**Figure 4:**
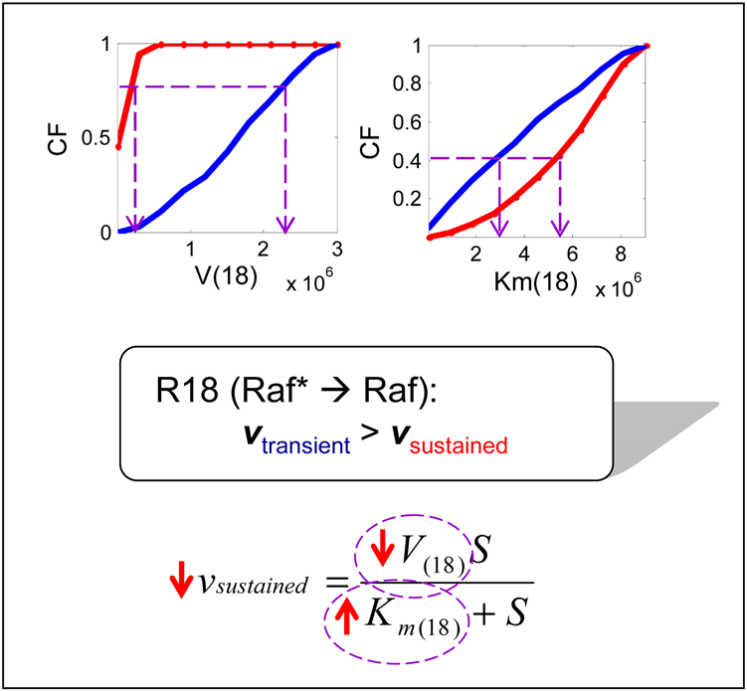
Analysis of the difference in reaction rates between the transient and sustained case for reaction 18 (R18: Raf→Raf*). The cumulative frequency (CF) distribution informs that the higher frequencies of larger Km18 and smaller V18 in the sustained case indicate a much slower reaction rate of R18 in the sustained case than in the transient case (i.e., v_s_<v_t_).

In taking a detailed look at the reactions, first, R14 and R16 are those that balance active Ras between an inactivated and activated state either by binding to GAP (to be finally converted to its inactive form (RasGDP)) or by binding to Raf (to further process the downstream pathway of active Raf). This activation of Raf becomes important for initiating the phosphorylation of MEK to MEKP through R19 (Note: reaction steps from R19 to R26 denote the phosphor-/dephosphorylation reactions in the MAPK module). The reverse reaction (R18) that dephosphorylates active Raf (Raf*) into its inactive form is also found to be a sensitive, controlling factor. We also find that those samples that show transient ERK activation tend to have smaller values of k14, V20, Km18, Km22 (and less so of Km25), but larger values in k16, k19, V18, and Km 24, Km26 (see **Figure S2**). For instance, the Ras dephosphorylation reaction rate (k14) is slower for the transient case than for the sustained one. Besides, higher frequencies of smaller Km18 values indicate that the affinity of active Raf to its specific enzyme PP2A is far more stable and stronger, resulting in a much faster Raf inactivation in the transient as compared to the sustained case. The higher frequency distribution of smaller Km22 also indicates faster MEKPP dephosphorylation in the transient case (**Figure 4**). Similarly, the larger k16 value expresses faster reaction activity in the transient case, and the distribution of larger values of parameter V18 indicates that R18 occurs much faster as well (**Fig. 4**). Lastly, larger constants Km23, Km24, and Km26 imply less active ERK phospho-/dephosphorylation reactions in the transient case, compared to the sustained case. These observations reveal that although the downstream signal to the ERK activation occurs much faster, it is ‘short-lasting’, thus producing a transient behavior. In contrast, for the sustained ERK response, active Raf (Raf*) lasts longer so that dephosphorylated MEKP and MEKPP have a higher chance to be re-activated, which will eventually produce a prolonged signal for ERK activation. Taken together, the association of both active Ras *and* Raf are crucial for controlling the response of ERK in the MAPK signaling cascade. Furthermore, reactions related to kinases and phophatases for both MEK and ERK are also shown to be important for distinguishing between transient or prolonged ERK activation as a consequence of EGF stimulation.

To examine the impact of specific pathway modules on differential ERK activities, we divided the entire EGFR pathway into three modules and only perturbed parameters that were involved in a particular subsystem. The results follow in the next section.

### Intermediate module reactions related to Ras and Raf are the most sensitive steps controlling the differential ERK response

For the first top-level module spanning from reaction step 1 to 8, we perturbed seven forward kinetic parameters (k1, k2, k4, k5, k6, k7, and k8) and three kinetic parameters of reverse reactions (kr1, kr3, and kr7) while fixing the other parameters at their nominal values. In examining the differentiating pathway parameters between the tolerable and intolerable group, parameter k1, i.e. the first reaction rate related to ligand-receptor binding, was found to be the most sensitive one. However, the top-level module parameter perturbations resulted in only transient responses of ERK activity. This suggests that parametric variations of the top-level module are unlikely to cause significant differences in ERK responses. A similar picture emerges for the third, i.e. MAPK module, where separate perturbation showed no sustained ERK activity pattern either. However, differential responses of ERK activity (transient or sustained behavior) were observed when the second or intermediate module was perturbed. This result was similar to those obtained with the whole pathway run (see **Figure S3**). Specifically, in the intermediate module, R14, R16, and R18 were the most sensitive reactions, which are all involved in determining the activation level of Ras and Raf. Consequently, these observations indicate that differentiation-reactions in the third module such as R20, R22, and R24–R26, which were discovered as sensitive reactions in the whole-pathway perturbation, seem to be strongly influenced by the reactions of the intermediate module rather than affecting differential ERK activity patterns on their own.

### What are the most informative reactions controlling the amplitude of transient ERK responses (i.e., L-T vs. H-T)?

In parallel to the case of transient vs. sustained ERK profiles, we continually investigated the differentiation-reactions that are instrumental for controlling the amplitude of ERK activity for the transient case. The result clearly shows that R18, i.e. the dephosphorylation of active Raf (Raf*), is no longer a sensitive reaction for the amplitude-differentiation between the low-transient and high-transient level (see **Figure S4**). However, R14 and R16, critical steps for Ras deactivation and Raf activation, respectively, remained sensitive factors in determining the amplitude variation.

### What are the most informative reactions controlling the extent of sustained ERK responses (i.e., L-S vs. H-S)?

We also searched for sensitive factors for the duration-differentiation pathway within sustained profiles. Here, not only did R14 and R16 turn out to be critical, but we also discovered that reactions that are specifically involved in the MAPK module became increasingly important, i.e., R19 (k19), R22(Km), R23(Km23), R24(V24, Km24), R25(Km25), R26(Km26) as shown in **Figure S5**. This indicates that phosphorylation and dephosphorylation of MEK and ERK play an important role in controlling differentially prolonged ERK phenotypes.

### How do changes in initial conditions and variations of the feedback regulation through active ERK impact the results?

**Figure 5:**
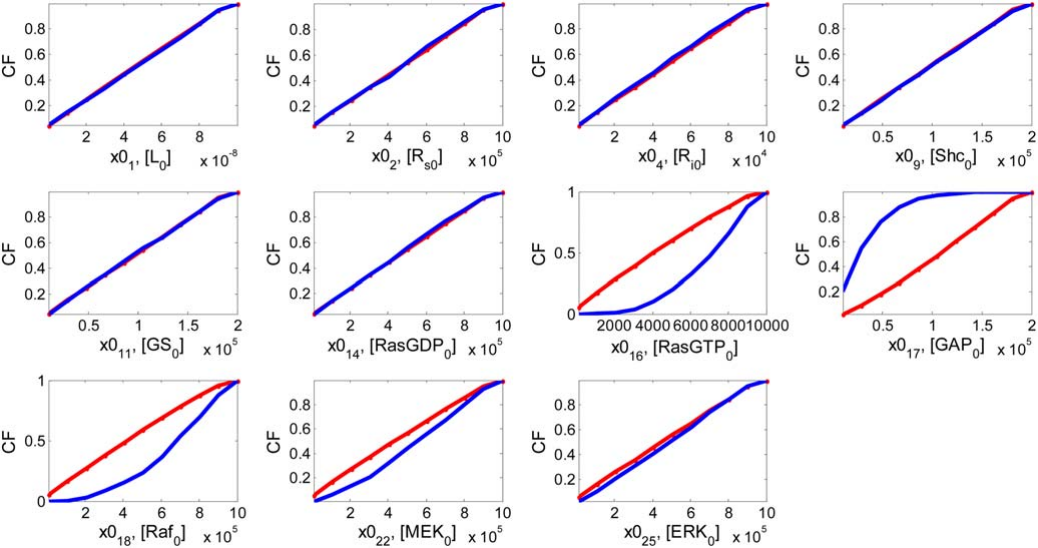
Results of perturbing multiple initial conditions. Solid lines in *red* and in *blue* represent tolerable and intolerable group, respectively.

First, we investigated which initial conditions are most influential in distinguishing differential ERK responses. By fixing parameters at their nominal values, a total of 11 initial conditions were perturbed at the same time. The ERK profiles all show transient behaviors with different amplitudes. **Figure 5** demonstrates that the initial concentrations of active Ras (RasGTP, x0(16)), GAP (x0(17)), and Raf (x0(18)) are the most sensitive ones that distinguish between tolerable and intolerable groups of samples with different amplitudes. These three protein molecules are key substrates involved in switching gears between Ras inactivation by binding to GAP (R14) and direct activation of the MAPK cascade by forming the Ras-Raf complex (R16). Specifically, the adaptor protein, GAP, is another limiting substrate in addition to active Ras in R14. Taken together, this indicates that not only the previously discovered kinetic parameters such as k14, k16, V18, and Km18, but also the initial concentrations of the substrate proteins involved in the corresponding reactions are crucial in determining the direction of downstream activation pathways.

Secondly, the extent of inhibitory feedback regulation of dissociation of the SOS complex, mediated by phosphorylated ERK, was varied across a range from zero feedback to 2 orders of magnitude of its original feedback strength when being multiply perturbed with the intermediate subsystems. Results showed no significant ERK response differences triggered by variations in the feedback parameter (k27, Km27) (compare **Figure S6** with **Figure S3**).

**Table 2** shows the correlation coefficients of how robust the parameters are for all three cases, plus variations of initial conditions.

### Does a single parametric global sensitivity approach strengthen or weaken our multi-parametric approach?

**Figure 6:**
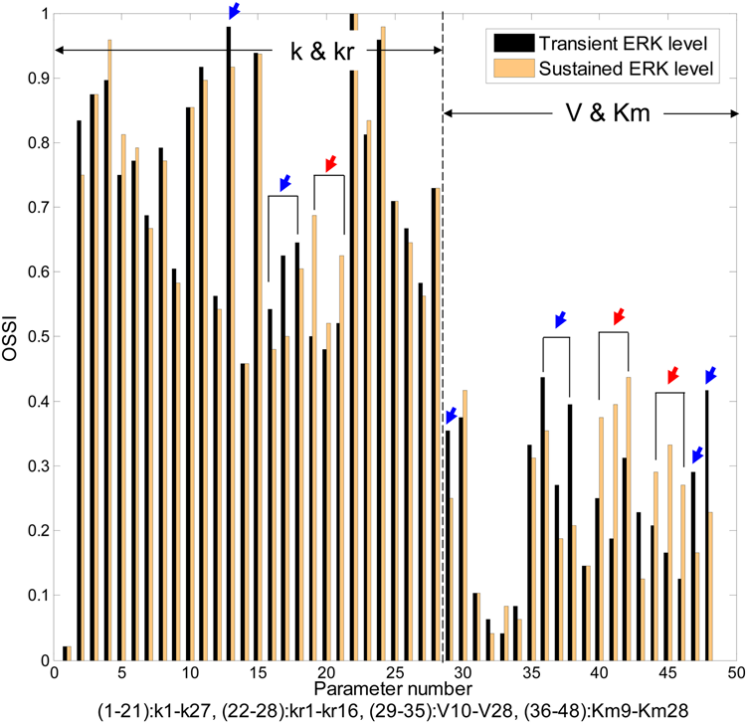
Comparison of the overall state sensitivity index between transient and sustained ERK levels. Parameter numbers from 1 to 21 denote all forward kinetic constants, k, 22 to 28 denote all reverse kinetic constants, kr, 29 to 35 denote all maximum reaction rates, V, in MM kinetics, and lastly, 36 to 48 denote all Michaelis constants, Km. *Blue* arrows indicate more sensitive parameters observed in the transient case, whereas *red* arrows indicate more sensitive parameters observed in the sustained case.

Lastly, we examined the effect of single parameter perturbation on overall state sensitivity and further compared the results with those from the multi-parametric approach. We perturbed one single parameter at a time while keeping the others fixed. Each parameter was perturbed with the maximum of (+/−) 1% and also (+/−) 50% variation around the nominal set of parameters. Note that the overall state sensitivity results were independent of the percentage variation. To identify controlling factors that contribute to transient or sustained ERK profiles, we selected two parameter sets for each case as an original parameter set, i.e., one set for transient behavior, another set for sustained ERK behavior. We replicated the perturbation simulation 100 times, ranked them for each run, and averaged their ranks by dividing the sum of ranks for each parameter with the total number of parameters (Np = 48). Thus, smaller OSS values reflect more robust parameters (**Figure 6**).

First, it is obvious that overall mass-action kinetic parameters such as k(2–21), kr (22–28) are more sensitive than MM kinetic parameters such as V (29–35) and Km (36–48). Secondly, it can be inferred that parameters that are most sensitive in orchestrating a transient ERK response have been initiated by intermediate-module reactions such as k14(13), k17(16), k19(17), k21(18), V10(29), Km9(36), Km10(37), and Km18(38). In contrast, parameters that are more sensitive for triggering a sustained ERK response are now strongly related to the MAPK module such as k23(19), k25(20), k27(21), Km20–Km22(40–42), and Km24–Km26(44–46). These are main reactions involved in phospho-/dephosphorylation of MEK and ERK by either kinases or phosphatases. Thirdly, the sensitive reactions of k23(19), k25(20), and k27(21) for the sustained case are interestingly those that are directly related to the ERK-induced inhibitory feedback loop. In addition, parameters Km27(47) and Km28(48) are less sensitive in the sustained case, implicating that R27 and R28 are more stably associated.

## Discussion

More mechanistic insights into key regulatory factors that control cellular phenotypic decisions are necessary to improve our understanding of cell biology in the normal and diseased state. Differential dynamics of the activation of the ERK-MAPK cascade is of importance in determining cellular responses such as cell proliferation and differentiation [6], [9]. It is, however, difficult to assess which factors in this cascade are crucial in controlling these differential cellular outputs because signal transduction mechanisms are inherently complex and highly nonlinear, and thus quantitative molecular interactions in the network can not be understood from structural pathway maps alone.

In this paper, applying *in silico* modeling, we have attempted to identify critical signaling factors in an EGF-induced MAPK signal transduction pathway. Such key factors can be protein molecules, kinetic parameters, or reactions as described in the result section. Using a Monte Carlo (MC) approach, all of the reaction parameters were systematically perturbed and investigated at the same time; similarly also for various initial conditions. To our knowledge, such a computational MAPK signaling cascade study, using a MC-based multi-parametric global sensitivity analysis, has not been done before to that extent, and without utilizing expert knowledge about the relative importance of its pathway components.

We have investigated three possible cases with regards to differential patterns of ERK activation, namely **(i)** transient vs. sustained, **(ii)** lowly transient vs. highly transient, and **(iii)** lowly sustained vs. highly sustained. Through our multiparametric analysis, we discovered that the intermediate module (rather than the top-level module or the MAPK module) dominantly controls ERK responses to being either transient or sustained. The key players regulating the distinct ERK behaviors were **1)** the binding-switch of Ras between GAP and Raf, **2)** Raf inactivation, and **3)** the initial conditions of RasGTP, GAP, and Raf. As a result of the fluctuation of these intermediate module molecules, MEK and ERK phosphorylations in the third, i.e., MAPK module, were sensitively co-regulating the differential ERK responses, but they did not contribute on their own. The comparisons of dominant reactions of these molecules in the three cases are summarized in **Table 3**.

**Table 3:** Comparisons of key dominant reactions for the three cases of two differential ERK activities.

**Case 1.: T vs. S**	**Transient dominant (T>S)**	**Sustained dominant (T<S)**
	R16, R18, R19, R22, R25	R14, R20, R23, R24, R26
**Case 2.: L-T vs. H-T**	**Lowly-transient dominant (L-T>H-T)**	**Highly-transient dominant (L-T<H-T)**
	R14, R20, R24, R26	R16, R19, R22, R25
**Case 3.: L-S vs. H-S**	**Lowly-sustained dominant (L-S>H-S)**	**Highly-sustained dominant (L-S<H-S)**
	R14, R20, R24, R26, R28	R16, R19, R22, R25

**Figure 7:**
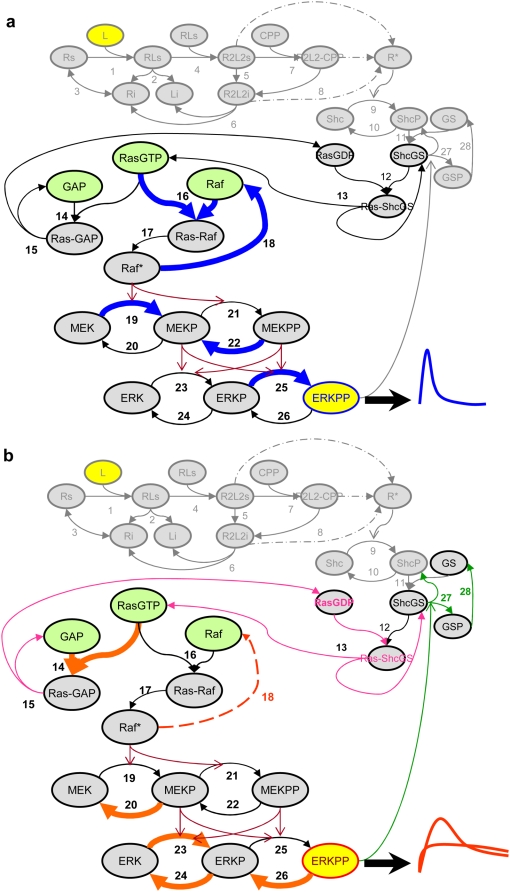
Mechanistic interpretations based on the multi-parametric global sensitivity analysis in Figs. 3 and 5. Shown is in (a) the dynamics of the transient ERK signaling response, and in (b) the dynamics of the sustained response. For the transient case in (a), the binding between RasGTP and Raf (R16) occurs faster than the binding reaction between RasGTP and GAP (R14), which further rapidly activates Raf. However, activated Raf is also quickly inactivated (R18) while it stimulates MEK phosphorylation (R19); in addition, activated MEK is also rapidly dephosphorylated (R22). These factors contribute to a faster, but short-lasting activation of ERK, thus producing a transient behavior. In contrast, for the sustained case in (b), the binding between RasGTP and GAP (R14) occurs faster, which increases the availability of RasGDP to bind to ShcGS complex so that the inactive Ras can be re-activated (R12 and R13) to continually stimulate its downstream. Meanwhile, Raf is still activated through R16 and R17. However, the inactivation of active Raf (R18) occurs much slower. In addition, the phospho-/dephospho-rylation reactions of ERK (R23, R24, and R26) are more active so that the signal can last longer within the MAPK module. Together, these factors result in a prolonged activation behavior of ERK. Note that initial protein concentrations of RasGTP, Raf, and GAP, found to be important, are depicted as *green* circles. In Fig. 7b, discovered by means of OSS, *green* arrows denote reactions R27 and R28, resulting from the inhibitory ERK feedback loop.

*How are these factors involved in regulating ERK dynamics?* First, faster activation of Ras and inactivation of Raf* (i.e., R16 and R18) governs transient ERK behavior. That is, a rapid interaction between Ras and Raf seems to accelerate a shorter signal sent to the downstream MAPK cascade, which results in a transient ERK peak. Consistently, the inactivation of Ras (R14; processed in parallel to R16) occurs much slower in a transient than in a sustained case. One of the potential reasons why Ras inactivation (i.e., R14) is slower in the transient case may be the availability of adaptor protein, such as GAP. The initial concentration of GAP or the ratio of availability between initial conditions of Ras, GAP, and Raf should therefore be considered as controlling factors, which may contribute to the differential ERK responses (**Figure 5**). Furthermore, activated MEK also experiences faster dephosporylation in the transient case than in the sustained case (R22). As the catalytic activation of ERK by MEKPP rapidly decreases, it may limit production of a prolonged activated ERK signal. These phenomena work inversely in the sustained case. **Figure 7** summarizes these suggested mechanisms for the differential dynamics of ERK signaling. Taken together, the dynamics of balancing between activation and inactivation of Ras and maintaining the stability of active Raf are central to controlling the distinctive ERK responses. We further note that the role of kinases and phosphatases involved in MEK and ERK phosphor-/dephosphorylation seems crucially important for the spectrum of ERK signaling responses as well.

Intriguingly, our results are in good agreement with reported computational and experimental findings. Recently, the importance of Ras dynamics has been further confirmed by investigating *in silico* dynamics based on the *in vivo* kinetics of ERK phosphorylation in PC 12 cells, where transient ERK activation depends on transient Ras activation with subsequent slow Ras-GAP activation [10]. However, to our knowledge, no anti-Ras strategies have managed to succeed in clinics yet [30]. One of the reasons is that Ras can utilize other effector signaling pathways such as Ral-GEF and PI3K to mediate cell transformation in different cell types. Although Ras FTIs (farnesyltransferase inhibitors) have been successful in post-translationally modifying Ras, it cannot block the transformation by mutationally-activated Raf and MEK [31]. Secondly, by applying an extended metabolic control analysis to an EGF-induced MAPK network, Hornberg et al. found that the activity of Raf controls all characteristics of the transient profiles of ERK phosphorylation, thus further confirming Raf's role as an oncogene [16]. Also, an experimental study by Fujioka et al., based on real-time monitoring of fluorescent probes in the MAPK cascade, supported a significant role for Raf in regulating ERK activity [24]. Thirdly, experimental work done by Adachi et al. showed that compound 5 (Cpd 5; a protein-tyrosine phosphatase (PTPase) inhibitor) caused prolonged ERK phosphorylation, which induced growth inhibition. However, when MEK inhibitors PD098069 or U0126 were given together with Cpd 5, both ERK phosphorylation and the growth inhibitory effect by Cpd5 were antagonized [32]; this implies that the level of MEK phosphorylation affects the distinct dynamics of ERK signaling. These early studies not only confirm our *in silico* findings about the role of Ras, Raf, MEK and ERK and their *cooperative* regulation mode of the EGF-induced MAPK downstream pathway, but also suggest a *multi-targeted* strategy (RasGTP, Raf *and* GAP in this case) if shutting-down such signaling cascades or network sub-modules would become a high value therapeutic goal [33].

Technically, the main advantage of our multi-parametric approach is the ease of discovery and interpretation of informative factors contributing to differentiation-pathways between separable output observations. It also provides a mechanistic view of the key factors involved while exact kinetic information is not required. However, one caveat is that parametric ranges for each parameter need to be carefully selected to cover the range of possible values; also, it fails to capture interactive effects between distant parametric factors on the structural map. For example, no feedback effect of active ERK to SOS (on differential ERK responses) was observed through our multi-parametric global sensitivity analysis. This may suggest that this feedback effect have been buffered by other dominant, intermediate reaction factors involved. In fact, one experimental work shows that the inhibition of ERK feedback to SOS was the least active feedback loop among multiple modes of negative feedback by phosphorylated ERK (refer to Figure 5 in [34]). At the risk of being computationally costly, the discovered informative factors may still need to be systematically examined to further verify that they are not involved in higher-order interactions. Regardless, the single parametric approach (OSS) supports our finding of a strong involvement of the intermediate module reactions in the transient case and confirms the marked role of MAPK module in the sustained case that we have seen with the multi-parametric analysis. However, OSS also yielded an intriguing result on its own in that the inhibitory feedback effect to SOS by active ERK in the MAPK module gained importance in controlling the sustained ERK profile (see **Figure 7(b)**). With respect to this, we note that it is true only for each single parametric perturbation, i.e., the inhibitory effect may not be active in the dynamic changes of multiple parameters as observed in our multi-parametric approach. Together, these observations from both multi- and single-parametric analysis support the need for further experimental validation.

There are other EGFR-downstream pathways that function in parallel to the MAPK pathway and which deserve our attention in future work. For example, Ras may have at least one more effector other than Raf such as PI3K (Phosphatidylinositol-3-kinase) [2], [35]. An adaptor protein, Grb2, one of the key proteins in the MAPK signaling cascade, is also an important co-mediator protein for the PI3K-Akt pathway which affects cell survival pathways. Thus, consideration of PI3K downstream may change cellular responses of ERK activation. It will be interesting to include such parallel or convergent pathways in the next iteration model and analyze how these additions, if at all, impact cellular phenotypic responses.

Ultimately, we intend to integrate this powerful molecular-level pathway analysis into microscopic-macroscopic-scale *spatial* modeling, specifically agent-based modeling [36]. This discrete-continuum or hybrid multi-scale modeling can help predict tissue-level tumor progression behaviors, which, in many cases, are attributed to molecular-level signaling cues [5]. Our multi-parametric approach described here will therefore advance multi-scale modeling platforms by exploring critical molecular elements involved in phenotypic decisions on a single-cell level [37]. As such, this combined approach may be applicable to facilitate anti-cancer target discovery and drug development.

## Supporting Information

Table S1 Correlation coefficients for the whole EGFR network. * T, a group of samples that show transient ERK activation; S, a group of samples that depict sustained ERK activation; L-T vs. H-T, samples of lowly transient ERK activation vs. samples of highly transient ERK activation; L-S vs. H-S, samples of lowly sustained ERK activation vs. samples of highly sustained ERK activation; Tol, samples of the tolerable group; iTol, samples of the intolerable group.
(0.08 MB DOC)

Figure S1 Schematic view of the MC simulation-based multi-parametric global sensitivity analysis.
(0.29 MB TIF)

Figure S2 Frequency distributions of parameters k14, V20, Km18, Km22, and Km25 in the first column, and those of k16, k19, V18, Km24, and Km26 in the second column for the whole pathway perturbation study. The red line and the blue line represent the sustained and the transient case, respectively. For instance, the frequency distributions for the transient case (blue line) in the first column show that smaller valued parameters are highly dominant whereas larger values are dominant for those parameters in the second column. These observations are exactly opposite for the sustained case (red line).
(0.32 MB TIF)

Figure S3 Results of cumulative frequency distributions of the multi-parametric sensitivity analysis for the intermediate module. Solid lines in red and in blue represent the sustained and transient case, respectively.
(0.30 MB TIF)

Figure S4 Results of cumulative frequency distributions between the lowly transient (L-T) and highly transient (H-T) case for the whole network. Solid lines in red and in blue represent the L-T and H-T case, respectively.
(0.54 MB TIF)

Figure S5 Results of cumulative frequency distributions between the lowly sustained (L-S) and highly sustained case (H-S) for the whole network. Solid lines in red and in blue represent the L-S and H-S case, respectively.
(0.53 MB TIF)

Figure S6 Results of cumulative frequency distributions of the multi-parametric sensitivity analysis for the intermediate module with the variation of feedback strength (k27, Km27). Solid lines in red and in blue represent the sustained and transient case, respectively.
(0.20 MB TIF)
