# Supplementary material for: Investigating Differential Dynamics of the MAPK Signaling Cascade Using a Multi-Parametric Global Sensitivity Analysis
Source: PLoS One. 2009 Feb 23;4(2):e4560. doi: 10.1371/journal.pone.0004560 (PMC2640453; doi:10.1371/journal.pone.0004560)
Supplement: Table S1 — Correlation coefficients for the whole EGFR network. * T, a group of samples that show transient ERK activation; S, a group of samples that depict sustained ERK activation; L-T vs. H-T, samples of lowly transient ERK activation vs. samples of highly transient ERK activation; L-S vs. H-S, samples of lowly sustained ERK activation vs. samples of highly sustained ERK activation; Tol, samples of the tolerable group; iTol, samples of the intolerable group. (0.08 MB DOC) [file pone.0004560.s001.doc]

| **Parameters** | **T vs. S** | **L-T vs. H-T** | **L-S vs. H-S** | **Initial Conditions** | **Tol vs. iTol** |
| --- | --- | --- | --- | --- | --- |
| k1 | 0.9975 | 0.9938 | 0.9956 | x_0_1 | 0.9997 |
| k2 | 0.9994 | 0.9983 | 0.9954 | x_0_2 | 0.9995 |
| k4 | 0.9992 | 0.9990 | 0.9912 | x_0_4 | 0.9996 |
| k5 | 0.9989 | 0.9945 | 0.9909 | x_0_9 | 0.9999 |
| k6 | 0.9984 | 0.9969 | 0.9929 | x_0_11 | 0.9997 |
| k7 | 0.9970 | 0.9982 | 0.9954 | x_0_14 | 0.9997 |
| k8 | 0.9989 | 0.9985 | 0.9944 | x_0_16 | 0.9196 |
| k9 | 0.9988 | 0.9991 | 0.9947 | x_0_17 | 0.7791 |
| k11 | 0.9983 | 0.9952 | 0.9921 | x_0_18 | 0.9435 |
| k12 | 0.9995 | 0.9952 | 0.9973 | x_0_22 | 0.9916 |
| k13 | 0.9997 | 0.9982 | 0.9825 | x_0_25 | 0.9994 |
| k14 | 0.9817 | 0.9823 | 0.9400 |  |  |
| k15 | 0.9972 | 0.9972 | 0.9962 |  |  |
| k16 | 0.9871 | 0.9860 | 0.9580 |  |  |
| k17 | 0.9996 | 0.9977 | 0.9899 |  |  |
| k19 | 0.9743 | 0.9759 | 0.8854 |  |  |
| k21 | 0.9994 | 0.9960 | 0.9959 |  |  |
| k23 | 0.9980 | 0.9968 | 0.9603 |  |  |
| k25 | 0.9891 | 0.9906 | 0.9913 |  |  |
| k27 | 0.9999 | 0.9988 | 0.9973 |  |  |
| kr1 | 0.9995 | 0.9984 | 0.9909 |  |  |
| kr3 | 0.9998 | 0.9992 | 0.9898 |  |  |
| kr7 | 0.9986 | 0.9989 | 0.9937 |  |  |
| V10 | 0.9990 | 0.9984 | 0.9927 |  |  |
| V18 | 0.4693 | 0.9960 | 0.9983 |  |  |
| V20 | 0.9479 | 0.9371 | 0.9229 |  |  |
| V22 | 0.9988 | 0.9985 | 0.9942 |  |  |
| V24 | 0.9919 | 0.9923 | 0.9613 |  |  |
| V26 | 0.9886 | 0.9837 | 0.9875 |  |  |
| V28 | 0.9986 | 0.9992 | 0.9853 |  |  |
| Km9 | 0.9980 | 0.9964 | 0.9919 |  |  |
| Km10 | 0.9989 | 0.9979 | 0.9953 |  |  |
| Km18 | 0.9571 | 0.9988 | 0.9910 |  |  |
| Km19 | 0.9968 | 0.9973 | 0.9955 |  |  |
| Km20 | 0.9980 | 0.9981 | 0.9936 |  |  |
| Km21 | 0.9996 | 0.9963 | 0.9964 |  |  |
| Km22 | 0.9673 | 0.9876 | 0.9497 |  |  |
| Km23 | 0.9878 | 0.9845 | 0.9666 |  |  |
| Km24 | 0.9956 | 0.9909 | 0.9868 |  |  |
| Km25 | 0.9917 | 0.9932 | 0.9871 |  |  |
| Km26 | 0.9906 | 0.9959 | 0.9668 |  |  |
| Km27 | 0.9998 | 0.9977 | 0.9967 |  |  |
| Km28 | 0.9994 | 0.9968 | 0.9946 |  |  |
