# Supplementary figures and images for: Investigating Differential Dynamics of the MAPK Signaling Cascade Using a Multi-Parametric Global Sensitivity Analysis
Source: PLoS One. 2009 Feb 23;4(2):e4560. doi: 10.1371/journal.pone.0004560 (PMC2640453; doi:10.1371/journal.pone.0004560)

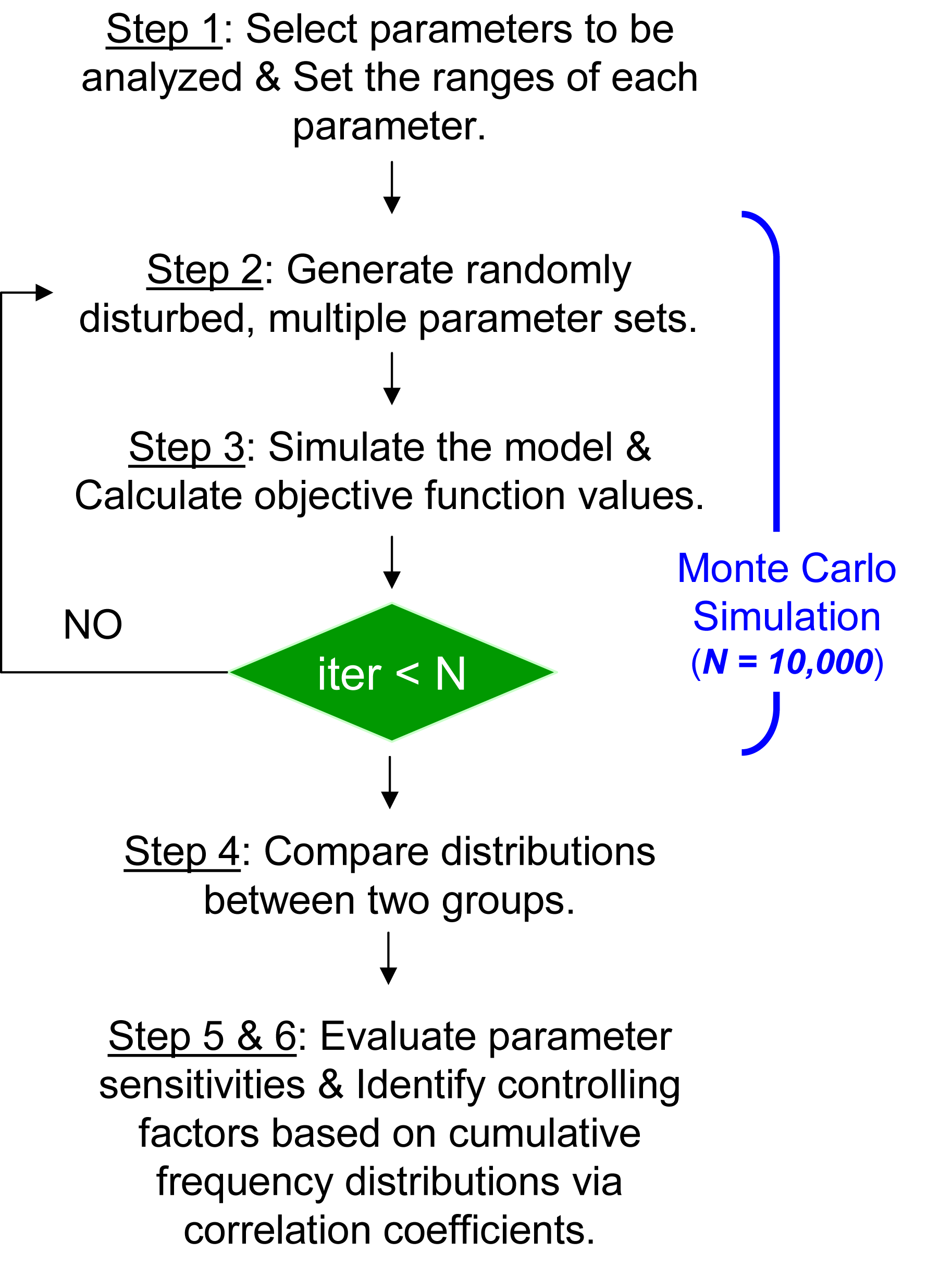

Supplement: Figure S1 — Schematic view of the MC simulation-based multi-parametric global sensitivity analysis. (0.29 MB TIF) [file pone.0004560.s002.tif]

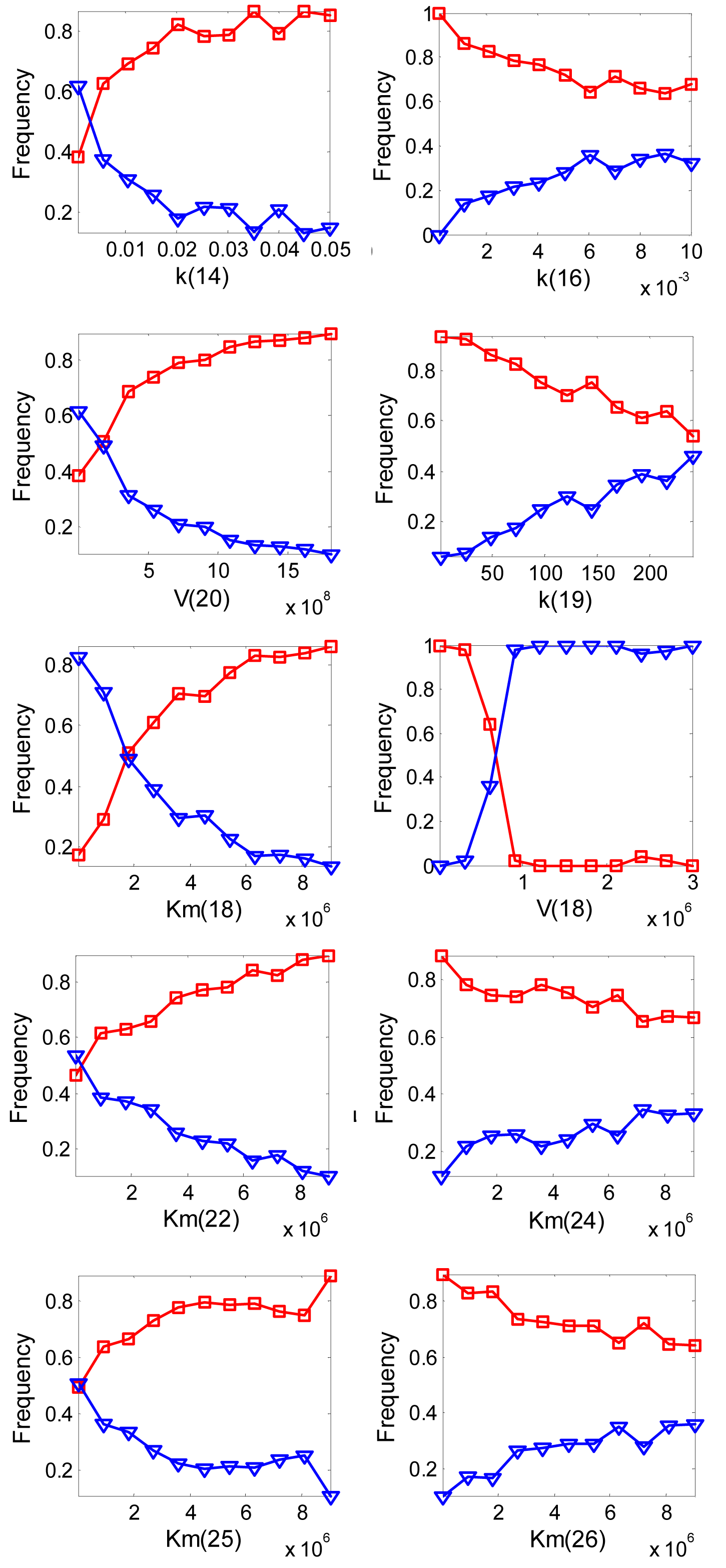

Supplement: Figure S2 — Frequency distributions of parameters k14, V20, Km18, Km22, and Km25 in the first column, and those of k16, k19, V18, Km24, and Km26 in the second column for the whole pathway perturbation study. The red line and the blue line represent the sustained and the transient case, respectively. For instance, the frequency distributions for the transient case (blue line) in the first column show that smaller valued parameters are highly dominant whereas larger values are dominant for those parameters in the second column. These observations are exactly opposite for the sustained case (red line). (0.32 MB TIF) [file pone.0004560.s003.tif]

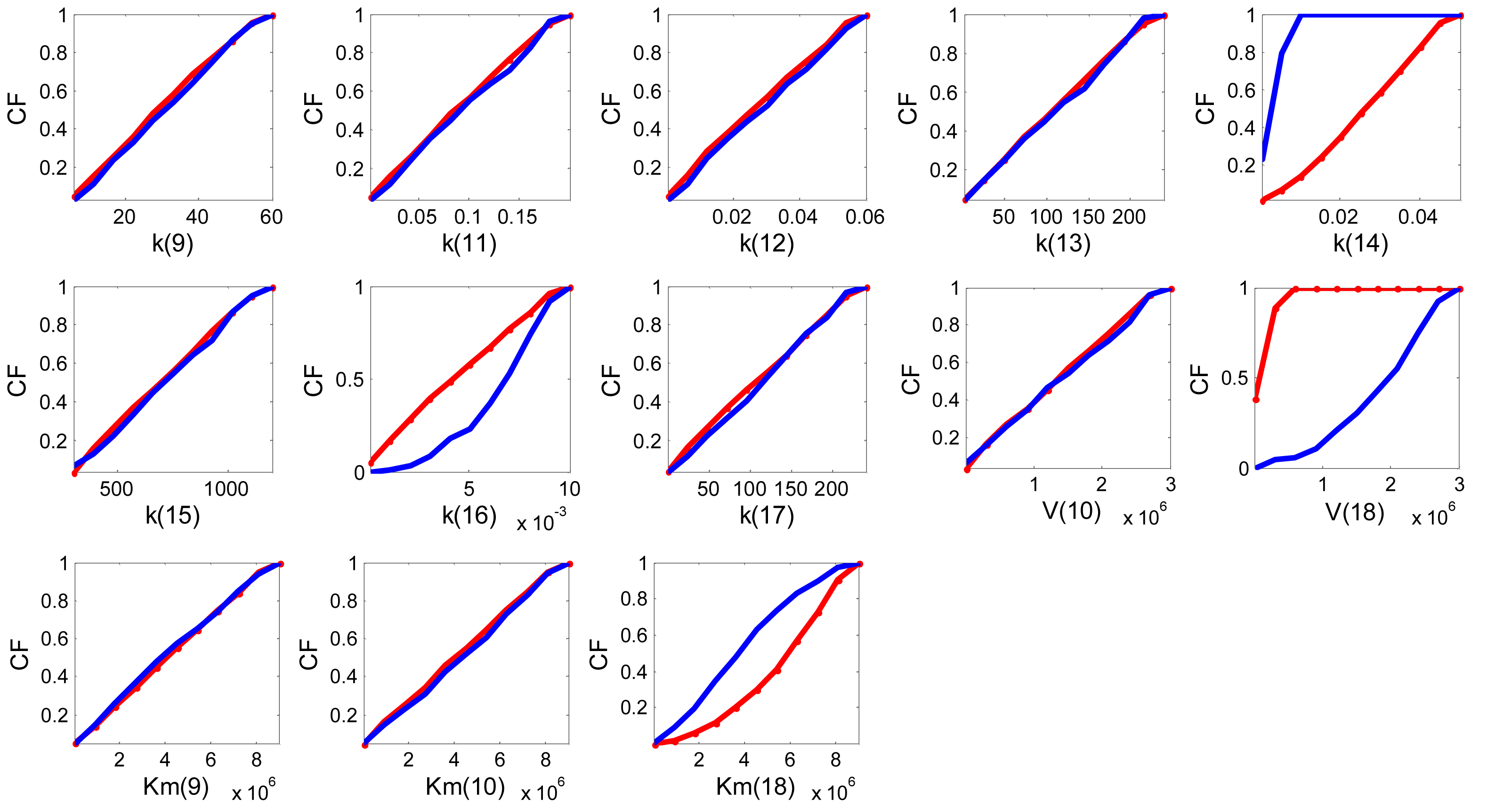

Supplement: Figure S3 — Results of cumulative frequency distributions of the multi-parametric sensitivity analysis for the intermediate module. Solid lines in red and in blue represent the sustained and transient case, respectively. (0.30 MB TIF) [file pone.0004560.s004.tif]

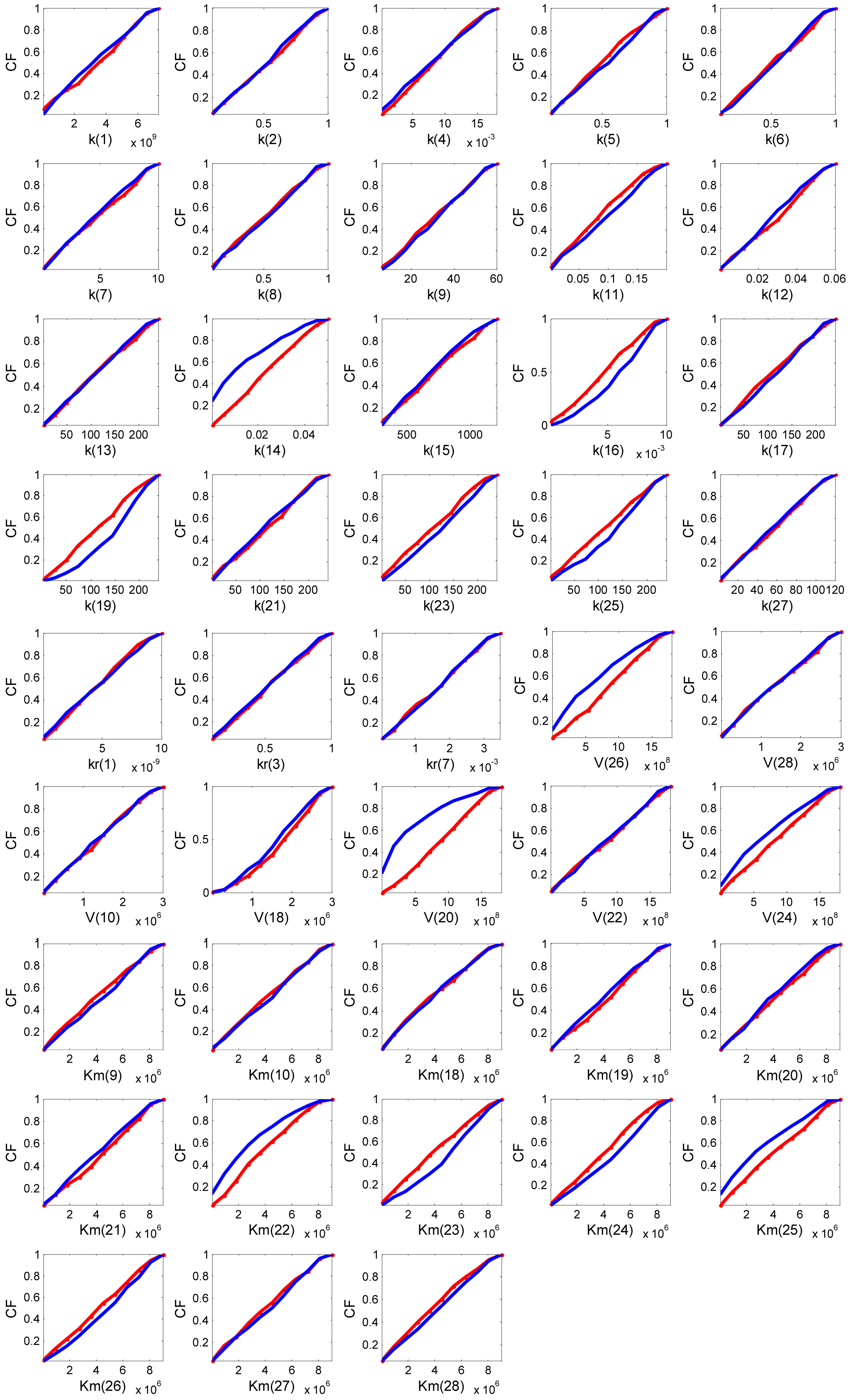

Supplement: Figure S4 — Results of cumulative frequency distributions between the lowly transient (L-T) and highly transient (H-T) case for the whole network. Solid lines in red and in blue represent the L-T and H-T case, respectively. (0.54 MB TIF) [file pone.0004560.s005.tif]

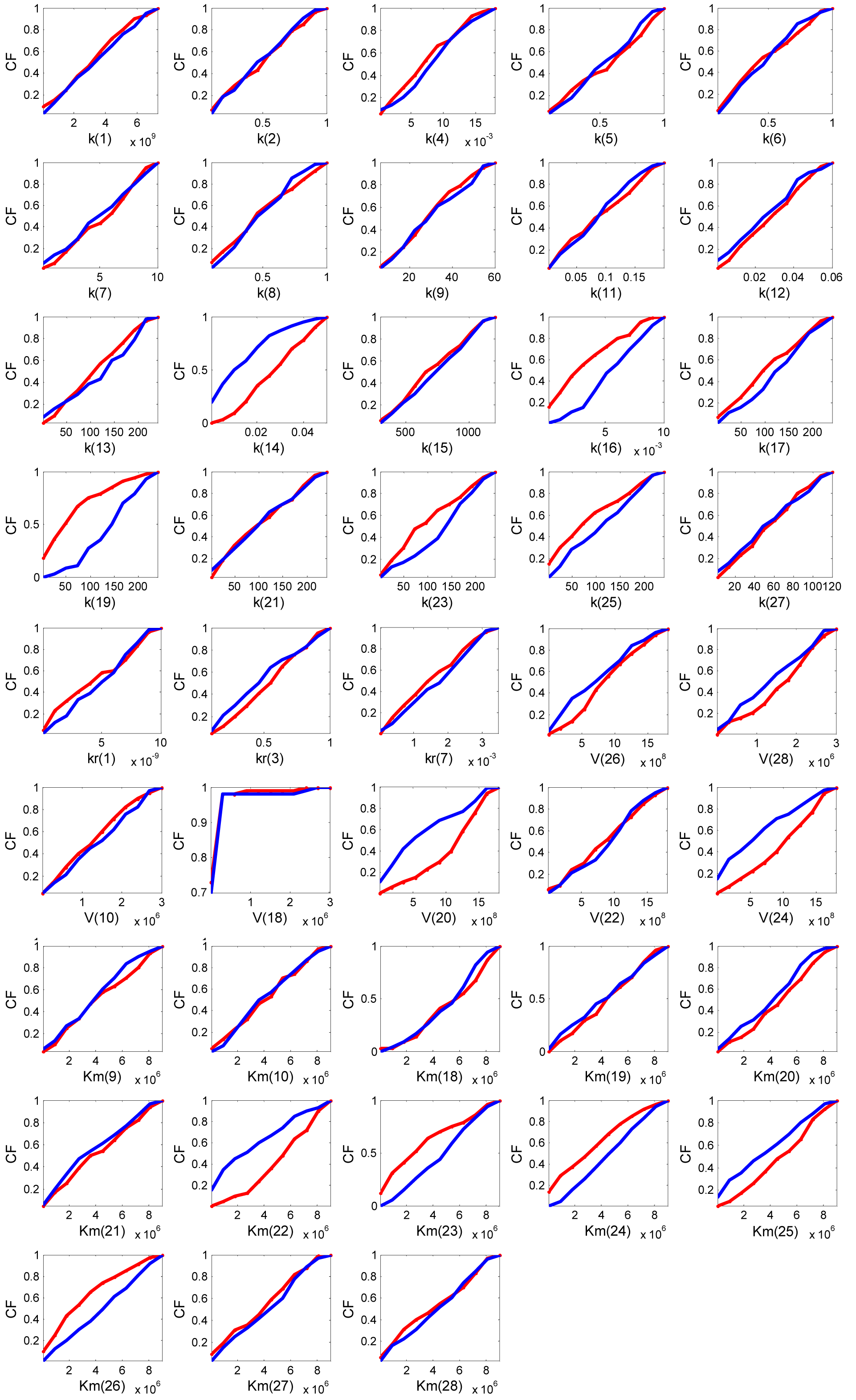

Supplement: Figure S5 — Results of cumulative frequency distributions between the lowly sustained (L-S) and highly sustained case (H-S) for the whole network. Solid lines in red and in blue represent the L-S and H-S case, respectively. (0.53 MB TIF) [file pone.0004560.s006.tif]

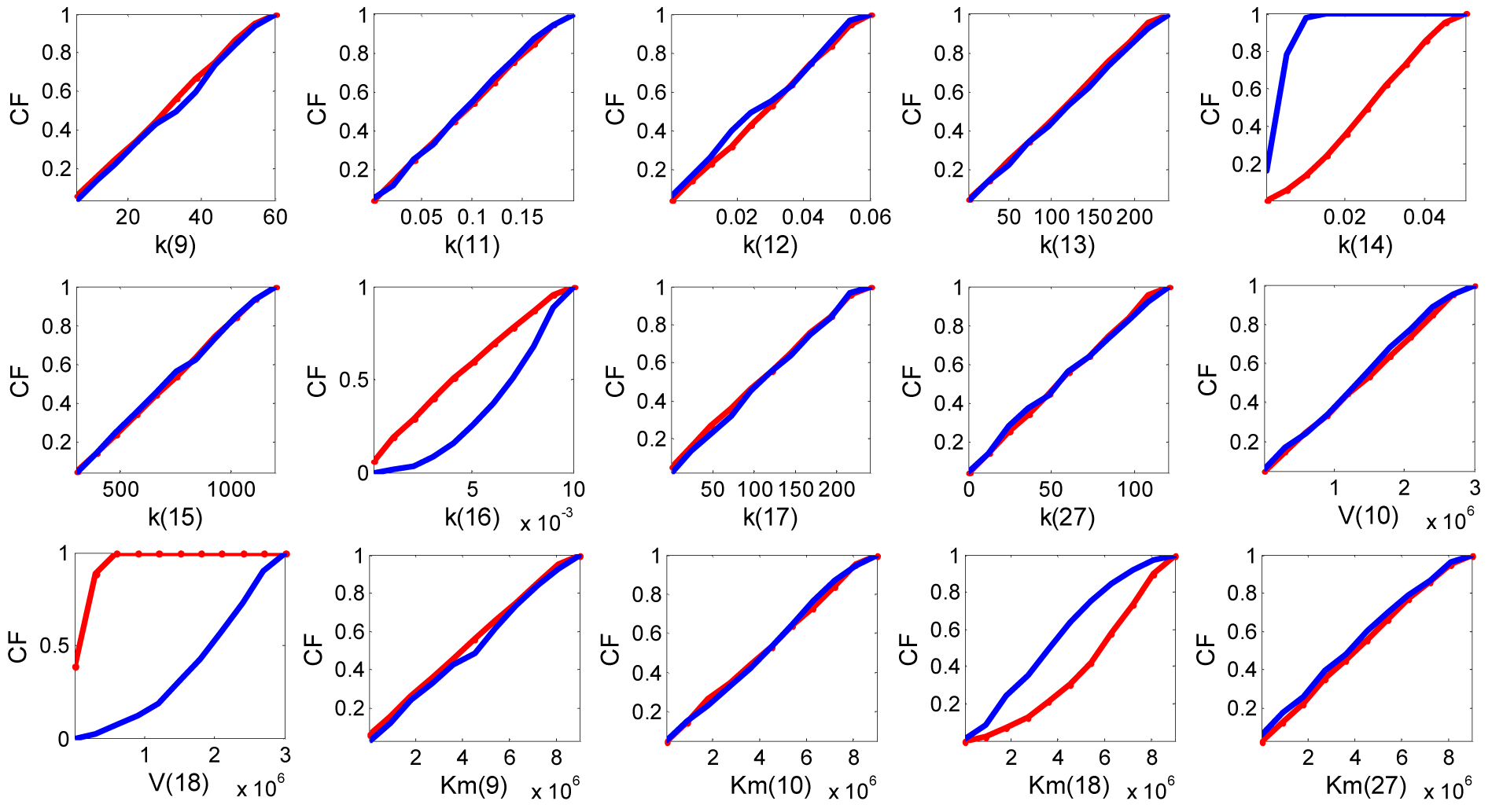

Supplement: Figure S6 — Results of cumulative frequency distributions of the multi-parametric sensitivity analysis for the intermediate module with the variation of feedback strength (k27, Km27). Solid lines in red and in blue represent the sustained and transient case, respectively. (0.20 MB TIF) [file pone.0004560.s007.tif]
